# Protein adsorption by nanomechanical mass spectrometry: Beyond the real-time molecular weighting

**DOI:** 10.3389/fmolb.2022.1058441

**Published:** 2023-01-05

**Authors:** Ivo Stachiv, Chih-Yun Kuo, Wei Li

**Affiliations:** ^1^ Department of Functional Materials, Institute of Physics, Czech Academy of Sciences, Prague, Czechia; ^2^ Department of Neurology and Centre of Clinical Neuroscience, First Faculty of Medicine and General University Hospital in Prague, Charles University, Prague, Czechia

**Keywords:** protein adsorption, molecule mechanical properties, molecular weight, protein detection, mass spectrometry, mass sensing

## Abstract

During past decades, enormous progress in understanding the mechanisms of the intermolecular interactions between the protein and surface at the single-molecule level has been achieved. These advances could only be possible by the ongoing development of highly sophisticated experimental methods such as atomic force microscopy, optical microscopy, surface plasmon resonance, ellipsometry, quartz crystal microbalance, conventional mass spectrometry, and, more recently, the nanomechanical systems. Here, we highlight the main findings of recent studies on the label-free single-molecule (protein) detection by nanomechanical systems including those focusing on the protein adsorption on various substrate surfaces. Since the nanomechanical techniques are capable of detecting and manipulating proteins even at the single-molecule level, therefore, they are expected to open a new way of studying the dynamics of protein functions. It is noteworthy that, in contrast to other experimental methods, where only given protein properties like molecular weight or protein stiffness can be determined, the nanomechanical systems enable a real-time measurement of the multiple protein properties (e.g., mass, stiffness, and/or generated surface stress), making them suitable for the study of protein adsorption mechanisms. Moreover, we also discuss the possible future trends in label-free detection and analysis of dynamics of protein complexes with these nanomechanical systems.

## 1 Introduction

**FIGURE 1:**
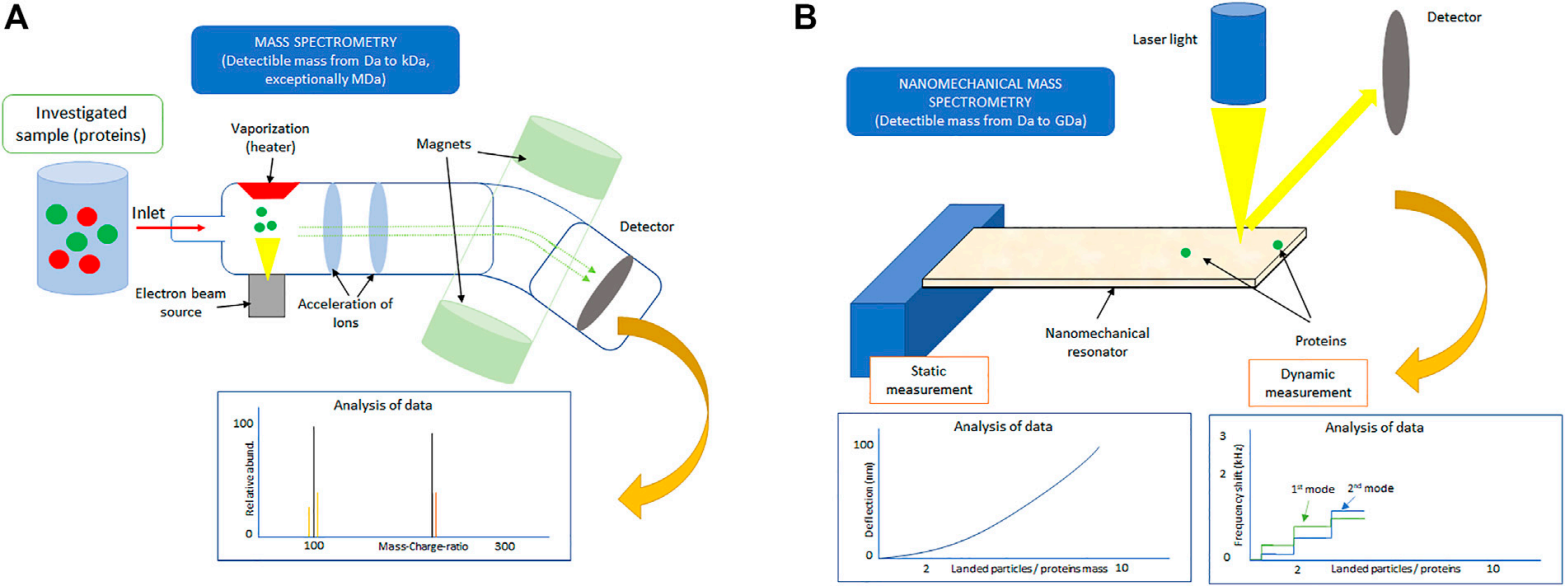
Sketch of **(A)** the conventional mass spectrometry and **(B)** nanomechanical mass spectrometry with commonly detectable analyte (protein) mass.

Proteins play a critical role in all living organisms. For example, they are responsible for intercellular transport and cell communication, or they also provide cells a given shape and mechanical resistance to deformation and contribute to DNA synthesis/replication (Satzer et al., 2015; Wingert et al., 2021). Moreover, many biological processes such as protein aggregation, transmembrane signaling, inflammation, or even the blood coagulation cascade are initiated by the adsorption/interaction of proteins with various (bio-) material surfaces (Deng et al., 2021). Hence, the understanding of protein adsorption mechanisms is of great importance to not only the fundamental science but also the majority of biomedical applications including orthopedic implants, stents, or ultrasensitive diagnostic tools (Talha et al., 2019). Up to date, many experimental methods to analyze protein adsorption have been developed (Deng et al., 2021). However, most of these methods either provide only the statistically averaged information on the single-protein adsorption (e.g., quartz crystal microbalance method), need a sophisticated experimental setup (e.g., X-ray photoelectron spectrometry), or require the knowledge of protein properties *a priori* own measurement (e.g., for surface plasmon resonance) (Kubiak-Ossowska et al., 2019). In contrast, mass spectrometers (MS) offer a high degree of sensitivity and selectivity; therefore, they have become a fundamental experimental tool used for the simultaneous identification and analysis of a larger number of proteins, often referred to as proteomics (Aebersold and Mann, 2003). MS utilize the ionization of the sample of interest which is then brought to the gas phase and subsequently analyzed by comparing the mass of individual ions obtained from the observed mass-to-charge ratio with the available protein database as also shown in Figure 1A (Johnson et al., 2020). Unfortunately, the necessity of performing destructive ionization of the investigated sample and especially for the large protein molecules to link the observed complex pattern with the relevant protein(s) is a formidable challenge that restricts the application of the conventional MS to only a relatively light protein molecules; that is, MS is limited to samples with mass ranging from Da to kDa (see Figure 1A) (Angel et al., 2012).

More than a decade ago, alternative methods to conventional MS that do not require ionization of the investigated sample were proposed (Eom et al., 2011; Sage et al., 2015). These methods identify studied protein(s) indirectly based on the observed changes in the dynamic and/or static response of the nanomechanical resonators (NRs) (Boisen et al., 2011; Hanay et al., 2012; Stachiv et al., 2012). We remind the reader that the static mode measurement is usually used to identify proteins that form a thin-layer film on the surface of a nanomechanical sensor (Stachiv et al., 2014), while the dynamic mode enables the identification of a single protein adsorbed in an arbitrary position on the NR (Naik et al., 2009). Because of its superior mass resolution (Jensen et al., 2008), the dynamic mode, that is, detection of the resonant frequency shift(s), is preferable in nanomechanical mass spectrometry. The commonly achievable mass sensitivities of static and dynamic mode measurement methods are illustrated in Figure 1B. The simplest and also the most often used dynamic mode measurement method models the protein molecule adsorbed on the surface of NR as a point particle (Dohn et al., 2007). Then, by measuring frequency shift in at least three (two) consecutive resonant frequencies of the cantilever (suspended) NR, the “protein” mass and its position of attachment can be determined (Stachiv et al., 2012). A very good agreement between theory and experiments enabled to confirm the validity of this multimode frequency shift measurement method (Schmid et al., 2010; Hanay et al., 2012; Sage et al., 2018; Ma et al., 2022). Nevertheless, this “simplest” method does not provide a sufficient amount of information needed to gain insights into the mechanisms of the protein adsorption on the surface of NR; that is, this method is primarily developed for nanoparticle mass sensing (Stachiv et al., 2012; Sage et al., 2018; Ma et al., 2022). Hence, there is an ongoing dispute on the application limits of the NRs, especially the multimode method, in the identification and analysis of a single or multiple protein(s) adsorbed on the resonator surface (Tamayo et al., 2013). Interestingly, recent studies have shown that NRs hold promise in the identification of even the larger masses (>GDa) (Dominguez-Medina et al., 2018; Stachiv and Gan., 2019; Stachiv et al., 2020; Erdogan et al., 2022). Moreover, there is a consensus among researchers that in order to gain more necessary information on the protein adsorption beyond its mass and position of attachment, a symbiosis of experiments with advanced theoretical modeling would be needed (Malvar et al., 2016; Stassi et al., 2019). In this study, we discuss the current findings, future trends, and perspectives in research and development of NRs for the identification of protein molecules beyond the simple molecular weighting. We also note that a systematic review of articles relevant to the considered topic of the present study was performed using the main electronic databases *via* Scopus, Google Scholar, and PubMed, which account for the majority of the journals focusing on the label-free detection and analysis of protein adsorption.

## 2 Nanomechanical mass spectrometry: From Da to GDa masses

The earlier studies on NR-based mass spectrometry have aimed to reach sensitivity comparable to that of commercial MS. We remind the reader that the achievable sensitivity of the NR depends on the resonant frequencies; that is, the higher operating frequencies of the NR yield the higher mass sensitivity (Ekinci et al., 2004; Gruber et al., 2019). To date, the most promising strategies to achieve the ultra-high operating resonant frequencies and, correspondingly, to enhance the mass sensitivity include the miniaturization of NRs (Yang et al., 2006; Xu et al., 2022), detection of the higher vibrational modes (Dohn et al., 2005; Ghatkesar et al., 2007) or, more recently, the non-linear oscillations (Yuksel et al., 2019; Lyu et al., 2020). It is noteworthy that during the past decade, the superb, that is, a Dalton, mass sensitivity has been experimentally demonstrated by using the one-dimensional carbon nanotube-based NR (Chaste et al., 2012), while in the same period of time the mass spectrometry community were trying to improve the capability of the conventional MS for measurement of larger (>MDa) masses including the protein complexes (Snijder et al., 2013). Hence, nowadays, the research attention of the nanotechnology community has also shifted toward the identification of larger masses, that is, in the GDa range, a goal that cannot still be reached by the conventional MS (Keifer et al., 2017).

Unfortunately, the unprecedented mass sensitivity of NRs has been confirmed for a large number of various nanoparticles (Yang et al., 2006; Jensen et al., 2008; Chaste et al., 2012), but for the protein molecules adsorbed on the resonator surface, the obtained results were inconsistent. Some studies showed that the point-mass approximation yields the accurately estimated masses (Naik et al., 2009; Hanay et al., 2012), whereas others revealed that this approximation fails to correctly determine the molecular mass (Gupta et al., 2006; Malvar et al., 2016). As a result, many theoretical models have already been developed to explain the effect of the bio-molecule adsorption on the NR frequency response (Dorignac et al., 2006; Tamayo et al., 2006; Lachut and Sader, 2007; Naik et al., 2009; Yi and Duan, 2009; Karabalin et al., 2012; Stachiv 2014). The majority of earlier studies suggested that during the molecule adsorption, the surface stress is generated causing changes in the resonant frequencies of NR, and, consequently, it can also result in the incorrectly estimated molecular mass (Dorignac et al., 2006; Yi and Duan, 2009). However, more recently, the surface stress effect has been proven to be insufficient to explain the inconsistency in the experimentally determined molecular masses (Karabalin et al., 2012; Lachut and Sader, 2012; Stachiv, 2014).

A different approach to gaining an understanding of the impact of biomolecules on the resonant frequencies was proposed by Tamayo et al. (2006). In their model, the adsorbate molecule was modeled as a cuboid with a given mass and stiffness. Then, based on the obtained computational results, they suggested that for larger masses or ultrathin resonators, where the adsorbed molecule and resonator thicknesses are of the same order, the molecule stiffness must be accounted when analyzing experimental data; that is, the point-mass approximation can result in the incorrectly identified molecular mass (Ruz et al., 2014). The validity of their model for mass sensing of larger molecules has been reinforced by comparing predictions with experiments carried out on gold nanoparticles and *E. coli* bacteria cells adsorbed on the cantilever-based NR (Malvar et al., 2016). It is noteworthy that the correctness of the point-mass approximation in MS of larger masses with arrays of NRs, each of distinct resonant frequencies obtained by altering the resonators’ dimensions, has also been successfully demonstrated (Sage et al., 2018). Whether the point-mass approximation or Tamayo’s model or even both models enable the accurate identification of larger masses was under extensive debate. Surprisingly, the application limits of both models have only been recently revealed (Stachiv et al., 2022a). In this work, they found that there exist three different sensing regimes, namely, ultra-light, light, and heavy, depending on the molecule-to-resonator mass ratio, for which the effect of the molecule properties (mass and stiffness) on the resonant frequency can be easily separated. Interestingly, they showed that the molecule stiffness plays an important role for NR in the ultra-light regime, that is, with the mass ratio <∼10^−3^, while for the light regime the point-mass approximation is always accurate, and, finally, for the heavy regime, that is, with the mass ratio >2 10^−2^, the molecule mass has a strong impact on the vibrational characteristic of NR, making the point-mass approximation inaccurate. The validity of these theoretical findings has been confirmed by comparing model predictions with the recent experimental results, and, as such, they can help to explain the observed inconsistencies in the molecule mass sensing. For instance, in the study of Malvar et al. (2016), the NR operates in the ultra-light regime, and correspondingly, the stiffness effect must be included when analyzing experimental data. Similarly, for Sage et al. (2018), the considered NRs operate in the light regime, enabling an accurate identification of the molecule mass even without accounting for its stiffness. In addition, the easily accessible procedure of mass sensing by NR in the heavy regime has recently been proposed (Stachiv et al., 2022b). Main findings of the aforementioned discussed key studies on mass spectrometry by the NR are, for the reader convenience, summarized in Table 1.

**TABLE 1:** Main findings of important experimental/theoretical studies focusing on the NR-based mass spectrometry in chronological order.

Study	Experimental setup/theoretical model	Main finding
Ekinci et al. (2004)	Pioneer theoretical study on the achievable sensitivity of NR	1) Expression for achievable mass sensitivity for different physical noise mechanisms was derived
		2) Achievable mass resolution of ∼1 Da was estimated
Tamayo et al. (2006)	One of the earliest theoretical studies describing possible mechanisms of molecule adsorption on NR	1) Developed model that accounts for both molecule mass and its stiffness
		2) Effect of molecule mass and stiffness on the resonant frequencies were elucidated
Gupta et al. (2006)	Important experimental study on the protein molecule adsorption on NR	1) In the first systematic experimental study, the effect of molecule thickness, its stiffness, and mass were investigated
		2) Impact of the protein molecule layer thickness on the resonant frequencies was explained
Dohn et al. (2007)	Combined theoretical and experimental study on particle mass sensing by NR	Procedure of a particle mass sensing (that is, point-mass approximation) has been developed for cantilever NR
Jensen et al. (2008)	One of the earliest experimental works on the carbon nanotube-based NR	Zeptogram sensitivity was achieved (∼.29 ± 05 zg) at a room temperature
Naik et al. (2009)	Pioneer work on the mass spectrometry by NR	Nanoparticles and also proteins were identified by NR with the resolution of ∼10 kDa
Stachiv et al. (2012)	Theoretical study on molecule mass sensing by NR	Easily accessible analytical expression, which enables determination of the molecule mass from the observed frequency shift, was derived
Chaste et al. (2012)	Experimental study on one-dimensional NR	Extraordinary, Dalton, mass sensitivity of 1-D NR was demonstrated
Karabalin et al. (2012)	Experimental and theoretical study	Theory which accounts for the surface stress effect on the resonant frequencies was developed and verified on NRs
Hanay et al. (2012)	Experimental study on mass spectrometry by NR	First experimental study, where single-protein mass sensing by NR was demonstrated
Sage et al. (2015)	Experimental work on nanomechanical mass spectrometry	Pioneer work, where the neutral particles were identified by NR.
Malvar et al. (2016)	Experimental study on mass and stiffness spectrometry by NR	Mass and stiffness mass spectrometry by NR was demonstrated for the first time, enabling to validate correctness of Tamayo’s model (2006)
Dominguez-Medina et al (2018)	Experimental work on larger molecule mass sensing by NR	A novel procedure of larger mass sensing (>100 MDa) by NR was developed and demonstrated
Sage et al. (2018)	Experimental study on larger molecule mass sensing by NR	Developed architecture of larger mass sensing (MDa) with NR using arrays of NR applicable to lap-on-a-chip
Yuksel et al. (2019)	Experimental work on non-linearly operating NR	Demonstration of the mass sensing by NR operating in a non-linear regime
Stachiv et al. (2020)	Theoretical work	Developed procedure of larger molecule mass sensing by NR operating in gaseous or aqueous solutions
Stachiv et al. (2022a)	Computational study	The effects of molecule size, mass, and stiffness on accuracy of determined mass and also the application limits the point-mass approximation used in mass spectrometry by NR were elucidated

We emphasize here that NR-based spectrometry proteomics is still not fully developed. Nevertheless, it can be expected that with the recent findings and continuous fast progress in NR-based spectrometry the full potential of these devices in proteomics will be achieved in a near future. We foresee that to fulfill a full potential of the nanomechanical systems in MS would require a significant simplification of the experimental apparatus, that is, these devices are still difficult to be fabricated and operated (Sansa et al., 2020), and development of the advanced computational models (Rocha et al., 2021; Xia et al., 2021) and approaches to nanomechanical mass sensing in also gaseous or aqueous solution (Urbanska et al., 2020). Furthermore, it is important to note that, as it was demonstrated by, for instance, Karabalin et al. (2012), Malvar et al. (2016), and Sader et al. (2018), the NR can already provide an additional information on the molecule adsorption than just the simple mass; that is, NR can be used to detect the generated surface stress and/or the molecule stiffness.

## 3 What other properties can be determined beyond the molecular weight?

It is a well-known fact that protein stiffness plays a crucial role in a large number of processes within living organisms (Senese et al., 2018; Li et al., 2021). For example, protein stiffness is responsible for the catalytic efficiency of many enzymes (Richard, 2019), and, additionally, even some mutation processes can be associated with relatively small changes in protein stiffness (Pfeifer et al., 2017). The mechanical stress contributes to the folding and unfolding of proteins, which is important in the modulation of a variety of cellular functions such as cell replication and metabolisms or ion transport (Eisner et al., 2018). Measurements of both the mechanical stress and protein stiffness are usually performed *in vitro* using either a combination of several experimental apparatus such as optical and/or magnetic tweezers combined with the atomic force microscope (Yang et al., 2020) or a highly sophisticated devices including the centrifuge force microscope (Yang et al., 2016), unconfined force testing machines (Su et al., 2019) or *in situ* TEM/SEM nanoindentation (Shen et al., 2020).

Nanomechanical sensors can detect numerous physical quantities such as mass, temperature, pressure, or force; therefore, they have been considered a suitable candidate for measurement of the mechanical properties and stress of the adsorbate protein molecule (a detailed discussion on nanomechanical sensors can be found in the review of Ruz et al. (2021)). The biomolecule adsorption-induced surface stress is usually measured using the static mode of the NRs, that is, by using the optical (Kidane et al., 2020) or resistive (Minami et al., 2021) measurement methods. Note that prior measurements of the functionalization of the surface of NR are necessary to immobilize the molecule of interest on the resonator surface (Zhang et al., 2018; Zhao et al., 2019). Once the molecule is adsorbed on the functionalized resonator surface, it generates surface stress, which, in turn, causes deformation of the NR. Hence, by detecting changes in the deflection of NR, the surface stress can easily be estimated (Boisen et al., 2011; Minami and Yoshikawa, 2021). Importantly, a theoretical model, which accounts for the molecule and effects of functional film viscoelastic properties on the adsorption-induced stress and/or strain, has only recently been developed (Minami et al., 2021). It is expected that this model will help researchers to gain insights into the mechanisms of the biomolecule adsorption-induced stress, and, correspondingly, the cellular functions could be studied.

Gil-Santos et al. (2010), in their pioneering work, demonstrated the extraordinary capability of carbon nanotube-based NR in mass sensing and stiffness spectrometry. They showed that by monitoring the nanotube vibrations in two directions, the adsorbate mass and its stiffness can simultaneously be determined with high accuracy, even for the protein stiffness, that is, the error in stiffness is within .1%. As mentioned previously, the first mass and stiffness spectrometry on larger molecules by means of NRs was realized in the study of Malvar et al. (2016). More recently, the same group showed that the ultra-high operating disk NRs may be used to retrieve other information on the protein, including the molecule hydration (Gil-Santos et al., 2020). The main drawback of these methods is the requirement of solving the complex inverse problem while taking into account the non-trivial molecule–resonator surface binding effects, molecule size, and stiffness (Malvar et al., 2016; Gil-Santos et al., 2020). Another limitation of this NR is the limited capturing and sensing area. This problem has already been solved by using an array of the high-frequency operating NRs (Sage et al., 2018; Lamberti et al., 2022). Gil-Santos et al. (2020) also showed that the resonant frequencies of the NR must be close to those of the investigated analyte (molecule). Only in this case, the analyte vibrational properties can be estimated. It is evident from the present discussion that this measurement would require the NRs with a tunable spectrum of the resonant frequencies (Liu et al., 2015). Frequency modulation by means of the piezoelectric effect (Kozinsky et al., 2006) or electrostatic actuation (Zhang et al., 2015) has been developed for frequency tuning of resonators.

Alternatively, the nanomechanical resonators with the NiTi shape memory alloy-based thin films have been shown having an extraordinary frequency tunability (Stachiv et al., 2017). This superb tunability is achieved by the diffusionless martensitic transformation in NiTi, while the high operating frequencies are due to the elastic substrate. It has also been demonstrated that NiTi-based NR can be used to measure mass, stiffness, and adsorption-induced surface stress (Stachiv and Sittner, 2018). This measurement utilizes a phase transformation in the shape memory alloys, which causes changes in the resonator stiffness; that is, at a low temperature NiTi is in the martensite phase with *E* ≈ 25 GPa, while at the high temperature NiTi transforms to the austenite phase with *E* up to 80 GPa and altering the interlayer stress, that is, the stress between NiTi and substrate can change more than four times (Stachiv et al., 2021). It can be easily expected that the shape memory alloy-based resonators would play a significant role in the next-generation nanomechanical mass spectrometers. These smart materials will enable resonator mechanical properties and interlayer stress to be adjusted based on the investigated analyte (protein molecule). In addition, shape memory materials can sustain a large recoverable deformation of more than 5% in the case of NiTi films (Juan et al., 2008). Hence, the shape memory alloy-based NRs can be also designed to change its shape before or during own measurements. We foresee that these resonators would enable manipulation of the molecule of interest simply by changing the resonator shape.

## 4 Nanomechanical mass spectrometry in fluids

Extraordinary high sensitivity of NRs can only be obtained in the vacuum, whereas in aqueous solution, which is relevant to the majority of biological systems and, therefore is also of emergent interests from the life science community, the mass sensitivity strongly degrades. Hence, by immersing the NR into fluid, the quality factor and mass sensitivity notably decrease, that is, sensitivity is usually > MDa (Eom et al., 2011). Burg et al. (2007) , in their pioneer study, suggested using the hollow resonator, where the measured molecule flows in the fluid inside the resonator. This resonator is then entirely operating in the vacuum. As a result, they achieved a mass sensitivity of tens of kDa. More recently, this technique has notably been improved, and the sensitivity of kDa has been demonstrated (Kim et al., 2016; De Pastina et al., 2018). Other approaches to mass sensing in fluids utilize measurement of quality factor changes (Stachiv et al., 2020). In this alternative approach, the changes in quality factor caused by the bound analyte are used to extract the analyte mass. Unfortunately, up-to-date measurement in gaseous or aqueous solutions has not reached sensitivity yet comparable to that of NRs operating in the vacuum. Nevertheless, it can also be expected that with further improvements and optimization of the hollow resonators, the Dalton sensitivity could possibly be reached.

Overall, we highlight here recent progress in nanomechanical mass spectrometry, current challenges, and possible future trends in research.
